# Changes in Body Surface Temperature Associated with High-Speed Treadmill Exercise in Beagle Dogs Measured by Infrared Thermography

**DOI:** 10.3390/ani11102982

**Published:** 2021-10-15

**Authors:** Maria Soroko, Wanda Górniak, Kevin Howell, Paulina Zielińska, Krzysztof Dudek, Maria Eberhardt, Patrycja Kalak, Mariusz Korczyński

**Affiliations:** 1 Institute of Animal Breeding, Wroclaw University of Environmental and Life Sciences, Chelmonskiego 38C, 51-630 Wroclaw, Poland; wanda.gorniak@upwr.edu.pl; 2 Institute of Immunity and Transplantation, Royal Free Hospital, Pond Street, London NW3 2QG, UK; kevin.howell4@ntlworld.com; 3 Department of Surgery, Faculty of Veterinary Medicine, Wrocław University of Environmental and Life Sciences, Plac Grunwaldzki 51, 50-366 Wroclaw, Poland; paulina.zielinska@upwr.edu.pl; 4 Faculty of Mechanical Engineering, Wroclaw University of Science and Technology, Wybrzeze Wyspianskiego 27, 50-370 Wroclaw, Poland; krzysztof.dudek@pwr.edu.pl; 5 Department of Reproduction and Clinic of Farm Animals, Faculty of Veterinary Medicine, Wroclaw University of Environmental and Life Sciences, Plac Grunwaldzki 49, 50-366 Wroclaw, Poland; maria.eberhardt@upwr.edu.pl (M.E.); 102726@student.upwr.edu.pl (P.K.); 6 Department of Animal Nutrition and Feed Management, Wroclaw University of Environmental and Life Sciences, Chelmonskiego 38C, 51-630 Wroclaw, Poland; mariusz.korczynski@upwr.edu.pl

**Keywords:** dog, exercise, treadmill, body surface temperature, infrared thermography

## Abstract

**Simple Summary:**

Heat exchange between the body surface and the external environment plays an important role in the regulation of body temperature in animals. Previous studies on the effect of exercise on the distribution of body surface temperature in dogs have been conducted on a variety of breeds and did not employ specific defined regions of interest. The aim of our research was to assess the influence of high-speed treadmill exercise on body surface temperature using infrared thermography in selected body regions of healthy Beagle dogs, taking into account gait and recovery time. The study was based exclusively on the Beagle breed, which presents short, uniform, and straight hair, conducting heat more readily from the skin. Statistical analysis indicated the highest temperature at the upper forearm and thigh, and the lowest on the croup, back, and neck. The peak surface temperature values in all examined areas were observed after the canter and the post-gallop walk, and the lowest were observed 2 h after exercise. Our study confirms that the body surface temperature of Beagle dogs is influenced by high-speed physical exercise on a treadmill as a result of muscle activity and changes in blood flow. The proximal forelimb and hindlimb were the most influenced by exercise.

**Abstract:**

Evaluation of body surface temperature change in response to exercise is important for monitoring physiological status. The aim of the study was to assess the influence of high-speed treadmill exercise on body surface temperature using infrared thermography (IRT) in selected body regions of healthy Beagle dogs, taking into account gait and recovery time. Thermographic images of the dogs were taken before exercise (BE), after walk (AW), after trot (AT), after canter (AC), just after second walk (JAE), 5 min after exercise (5 AE), 15 min after exercise (15 AE), 30 min after exercise (30 AE), 45 min after exercise (45 AE), and 120 min after exercise (120 AE). Body surface temperature was measured at the neck, shoulder, upper forearm, back, chest, croup, and thigh. Statistical analysis indicated the highest temperature at the upper forearm, shoulder, and thigh, and the lowest on the croup, back, and neck. The peak values of surface temperature in all ROIs were at AC and JAE and the lowest at 120 AE. The study demonstrated that body surface temperature was influenced by high-speed physical exercise on a treadmill and IRT was a viable imaging modality that provided temperature data from specific body regions. The proximal forelimb and hindlimb were the most influenced by exercise.

## 1. Introduction

Heat exchange between the body surface and the external environment plays an important role in the regulation of body temperature in homeothermic animals [1]. A typical dog produces approximately 1 cal/kg/h of heat from the basal metabolic rate, reflecting the metabolism of all body cells at rest, which is sufficient to maintain a proper internal body temperature [2]. During exercise, about 70–80% of the energy produced by the working muscles is converted to heat, and its excess is removed from the body through the respiratory system and the associated evaporation function [3].

During exercise, the dog is capable of increasing its cardiac output by 74–200% and can increase its carotid blood flow by 500% [4], which optimises blood flow to regions of maximal heat exchange. Although a small amount of the heat produced by the working skeletal muscles is passively conducted by surrounding tissues to the outer skin, the majority of this heat is transferred by convection through the venous blood flowing from these muscles, and then directed to superficial veins to elevate skin surface temperature [5]. Therefore, the evaluation of body surface temperature change in response to exercise is important for monitoring physiological status and the factors limiting performance during physical activity [6]. Physiological response to exercise has been largely examined through recording rectal temperature, which requires invasive contact with the animal [7,8].

Infrared thermography (IRT) is a non-invasive technique that measures infrared radiation emitted from the body surface, providing a pictorial representation of body temperature in animals [9]. Several studies found that IRT may be a useful tool in assisting diagnosis of diseases affecting the canine locomotor system [10,11,12] or in the evaluation of emotional stress [13]. Elevated temperature can reflect the presence of inflammation in underlying tissues, or where blood flow is increased or decreased owing to a clinical abnormality. Reproducible thermal body patterns have been identified in healthy canines [14] with symmetrical distribution between both sides of the body [15]. The warmest body surface temperatures have been reported at the torso and hindquarters, with surface temperature distribution across the limbs giving rise to proximal areas warmer compared with distal parts [16]. Studies based on different dog breeds concluded that the biggest impact on body surface temperature was the type and color of hair coat [6,14,16].

The influence of exercise on body surface temperature distribution in dogs has been studied with IRT, where areas of the neck, shoulder, back, and thigh significantly increased in temperature after a treadmill session [6]. However, that study included different breeds, which introduced variability in surface temperature due to the different thickness and density of hair coat. Moreover, the study did not employ specific defined regions of interest.

The aim of our research was to assess the influence of high-speed treadmill exercise on body surface temperature using IRT in selected body regions of healthy Beagle dogs, taking into account gait and recovery time. The hypothesis of the study was that body surface temperature in specific body regions increases in response to high-speed exercise on the treadmill. The study was based exclusively on the Beagle breed, commonly used in canine thermography studies [14], which presents short, uniform, and straight hair, conducting heat more readily from the skin compared with hair that lays flat against the surface of the body [16].

## 2. Materials and Methods

Dogs qualified for the research were subjected to standard procedures without any harm or discomfort, thus the study did not require the consent of the Local Ethical Commission for Animal Experiments at the Institute of Immunology and Experimental Therapy of the Polish Academy of Sciences in Wroclaw, Poland (no 080/2020/P1, Act of 15 January 2015 on protection of animals used for scientific or educational purposes).

### 2.1. Animals

The study included nine clinically healthy Beagle dogs (four females and five males; age 4–17 years; mean body weight 14 ± 1.5 kg) with no history of lameness or musculoskeletal pathology, with similar daily physical activity levels. All dogs underwent a full physical examination to exclude possible clinical abnormalities. The dogs were owned by Wroclaw University of Environmental and Life Sciences, and were housed in individual pens (140 × 200 cm) lined with wood shavings. They were provided with daily exercise and were fed twice daily, with ad libitum access to water. The study was conducted in the research room of the Department of Internal Medicine and Clinic of Diseases of Horses, Dogs, and Cats, where dogs were familiarized with the treadmill exercise. Exercise on a treadmill of a similar type and duration as used in the research protocol was performed for 3 months preceding the study.

### 2.2. Treadmill Exercise and Infrared Thermography Examination

All dogs were prepared for the study according to the protocol for thermographic examination in veterinary medicine [17,18]. The study was carried out over 1 day on dogs, which were without any physical activity on the day of the study and were groomed one hour before their session on the treadmill. During the experiment, the dogs were not fed 12 h prior to exercise to ensure that they were in a postabsorptive state and had access to water provided ad libitum after finishing work on the treadmill. Prior to exercise, each dog underwent 20 min of acclimatization in the research room. The mean ambient temperature in the room, as well as in the examination facility, was maintained at 20 ± 2 °C with humidity of 60% (without major fluctuations). The doors of the examination room remained closed during both the acclimatization period and during treadmill exercise. The ambient temperature in the examination room was measured by a TES 1314 thermometer (TES, Taipei, Taiwan).

Dogs were exercised on a dogPACER model LF3.1 electric treadmill (dogPACER™, Piaseczno, Poland) for 35 min, consisting of walking (10 min), trot (10 min), canter (10 min), and second walk (5 min), and the procedure ended with 120 min of recovery time. The speed of the treadmill was the same for all dogs, allowing a comfortable active walking, trotting, and cantering speed, with the walking speed for all dogs being 2.5 km/h, the trotting speed for all dogs being 4 km/h, and the cantering speed for all dogs being 8.5 km/h. To standardize the exercise protocol, the treadmill acceleration, speed, and exercise duration were computer-controlled.

For thermographic examination, a VarioCam HR infrared camera (uncooled microbolometer focal plane array; resolution, 640 × 480 pixels; spectral range, 7.5–14 mm; InfraTec, Dresden, Germany) was used. Thermographic images of both sides of each animal were performed before exercise (BE), after walk (AW), after trot (AT), after canter (AC), just after second walk (JAE), 5 min after exercise (5 AE), 15 min after exercise (15 AE), 30 min after exercise (30 AE), 45 min after exercise (45 AE), and 120 min after exercise (120 AE). Both sides of the body were imaged at a 90° camera angle, from a distance of approximately 2 m from the treadmill, to ensure that the entire body was included in the image. The thermal imaging camera was set at an emissivity value (ɛ) of 1 [15]. All thermographic images before and after exercise on the treadmill were performed in the examination room. The imaging at 120 AE was performed in a separate room, where the dogs were kept in individual pens, with similar environmental conditions to the examination room. The ambient temperature in the separate room was measured by a TES 1314 thermometer (TES, Taipei, Taiwan).

**Figure 1:**
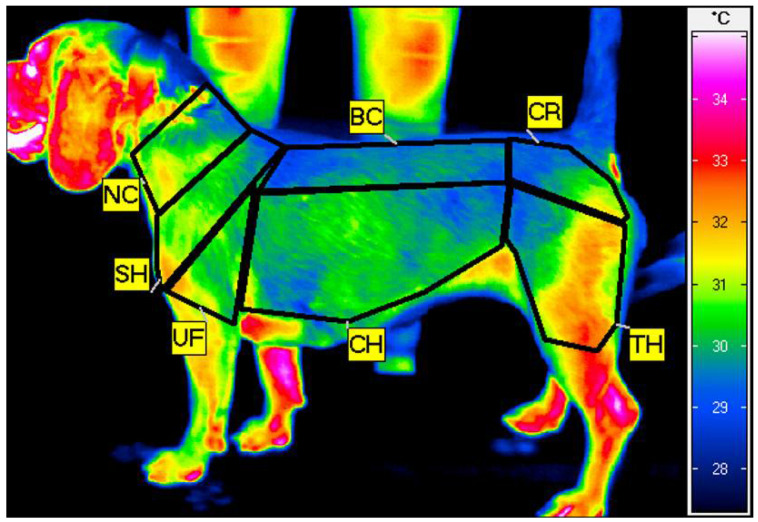
Example thermogram of the left side of the dog taken just after training on the treadmill with the seven regions of interest (ROIs) indicated: neck (NC), shoulder (SH), upper forearm (UF), back (BC), chest (CH), croup (CR), and thigh (TH).

The thermograms were analyzed using IRBIS 3 Professional software (InfraTec, Dresden, Germany). On each thermographic image, seven regions of interest (ROIs) were positioned, standardized by the width of the major muscle groups: the neck (NC), encompassing the serratus ventralis cervicis muscle; the shoulder (SH), encompassing the infraspinatus and supraspinatus muscles; the upper forearm (UF), encompassing the triceps brachii muscle; the back (BC), encompassing the longissimus muscle; the chest (CH), encompassing the chest muscles; the croup (CR), encompassing the gluteal muscles; and the thigh (TH), encompassing the quarter muscle. From each ROI, mean ROI temperature (Tavg) and standard deviation (SD) were calculated (Figure 1).

### 2.3. Statistical Analysis

Measurements of the left side of the body were used for analysis, as there were no statistically significant differences in Tavg between both sides of the body. The homogeneity of variance was checked with the Brown–Forsyth test and the Levene test. The normality of the temperature distribution was verified by the Kolmogorov–Smirnow test with the Lilleforce correction and the Shapiro–Wilk test. One-way analysis of variance (ANOVA) and multiple comparison tests (Tukey’s post hoc) were used to compare the temperatures in groups differing in ROI location or exercise timepoint. Initially, it was confirmed that the measured temperatures in each of the studied groups had a normal distribution and the same variances (*p* = NS, Shapiro–Wilk). Throughout the study, *p* < 0.05 was adopted as the critical level of significance. The TIBCO Statistica^®^ software package (v. 13.3.0, TIBCO Software Inc., Palo Alto, CA, USA) was used for all calculations.

## 3. Results

Statistical analysis confirmed a significant effect on temperature of high-speed treadmill exercise at all ROIs. There were no significant differences in surface temperature between the seven body regions at BE (*p* = 0.057). Table 1 presents the mean body surface temperature in the seven ROIs measured before treadmill exercise, immediately after each exercise phase, and during recovery time. There were significant body surface temperature differences between each stage of exercise and recovery time.

**Figure 2:**
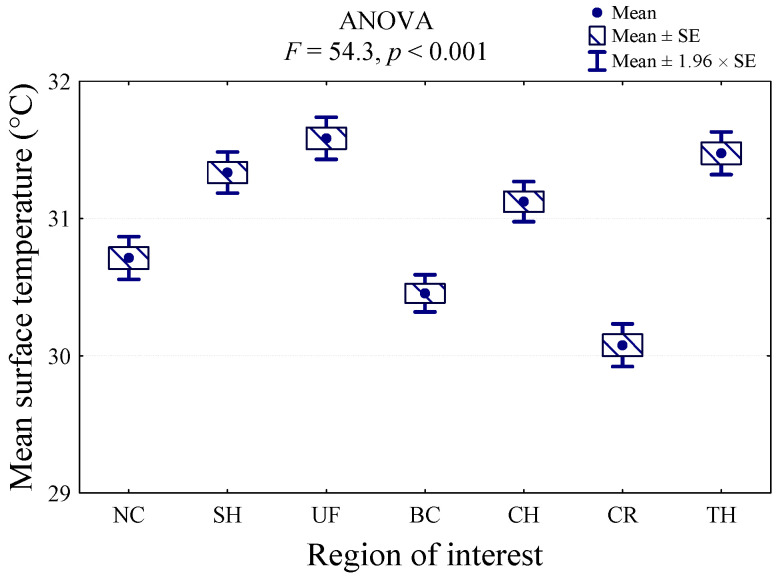
Comparison of average body surface temperature at seven regions of interest (ROIs): croup (CR), back (BC), neck (NC), chest (CH), shoulder (SH), thigh (TH), and upper forearm (UF) measured before treadmill exercise, immediately after each exercise, and during recovery time in nine dogs.

ANOVA indicated a significant effect of ROI on body surface temperature (*p* < 0.001; F = 54.3). The value of the analysis of variance statistics (F = 54.3) and the corresponding significance level (*p* < 0.001) indicate that the mean temperature of at least two regions differs significantly. The highest surface temperature was recorded in the UF region (31.6 °C), and it was significantly higher than the temperatures in the NC, BC, CH, and CR regions. The temperature was similar in the TH (31.5 °C) and SH (31.3 °C) regions. The lowest body surface temperature was indicated in CR (30.1 °C), and it was lower than all the other regions (*p* < 0.001). Furthermore, the BC region presented the low body surface temperature (30.5 °C), which did not differ significantly only from the NC region (30.7 °C) (Figure 2).

**Figure 3:**
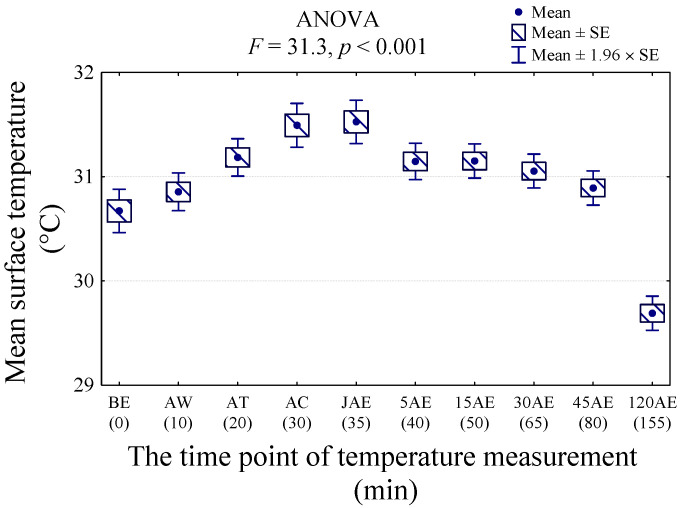
The average temperature (Tavg) of all regions of interest (ROIs) measured before exercise (BE), after walk (AW), after trot (AT), after canter (AC), after second walk (JAE), 5 min after exercise (5 AE), 15 min after exercise (15 AE), 30 min after exercise (30 AE), 45 min after exercise (45 AE), and 120 min after exercise (120 AE) in nine dogs.

Statistical analysis showed higher values of body surface temperature of all ROIs at AC and JAE, and the lowest temperatures at 120 AE (*p* < 0.001; F = 31.3) (Figure 3).

Compared with BE, multiple comparisons indicated a statistically significant increase in body surface temperature of all ROIs at timepoint AT, persisting until JAE, and returning to the initial temperature level at 5 AE (Table 2). The highest body surface temperatures were at timepoints AC and JAE (31.5°C). The average body temperature of the dogs before the exercise was 1 °C higher than the temperature after 120 min of recovery time (120 AE) (*p* < 0.001).

## 4. Discussion

Our study indicated that the body surface temperature of Beagles was influenced by high-speed physical exercise on a treadmill, confirming the results of previous research in canines [6,15]. A significant temperature increase at all ROIs occurred after trot and lasted up to 15 min after exercise (15 AE), suggesting that muscle work during the intensive phase of the exercise generated a considerable amount of heat. Furthermore, the subsequent vasodilation of the superficial vasculature and the distribution of the blood had an effect on the increase in body surface temperature.

Moreover, the results obtained suggested the highest surface temperature in all the studied body regions was after canter (AC) and just after exercise (JAE) on the treadmill. Similar results were reported by Rizzo et al. [6], where the highest body surface temperature in all measured regions, including the neck, shoulder, ribs, flank, back, internal thigh, and eye, was after the intensive phase of canter. Greyhounds after racing also demonstrated a significant increase in body surface temperature overlying the musculus gastrocnemius when measured with IRT [15]. This could be associated with an increase in muscle blood flow to meet the increased oxygen demand during exercise. As the internal temperature begins to rise, the vasodilatory response is also activated to transfer the heat generated in the muscles through the skin to the environment.

A study based on sport dogs, which measured core body temperature with ingestible sensors and ear temperature with IRT, reported temperature returning to resting levels 60 min after exercise [19]. In our study, however, the surface temperature in all regions was not significantly elevated above pre-exercise levels beyond the cessation of exercise on the treadmill.

In a study of horses working on a treadmill, there was no significant difference in body surface temperature obtained before exercise and beyond 45 min after the exercise was stopped [20]. The more rapid cooling of the body surface of dogs after exercise in our study would be consistent with their larger surface area to volume ratio than that of the horse, facilitating more efficient heat exchange with the environment [21].

Our data suggest a further decrease in body temperature in all ROIs by around 1 °C compared with BE two hours after exercise (120 AE). In canines, heat loss via respiration (panting) contributes to around 25% off all body heat loss at rest when the ambient temperature is below body temperature. This evaporative heat loss is used to cool blood flowing along the respiratory and breathing surfaces including the throat, mouth, tongue, and nose [22]. Panting is closely regulated by body temperature and occurs at rest, typically as air temperature increases to 30 °C, or with exercise, even at neutral to cool ambient temperatures [23]. For example, hunting dogs during a 15 km run with a rectal temperature of 41.2 °C lost 25.1% of heat production by respiratory evaporation, while domestic dogs, with a rectal temperature of 39.2 °C, lost 49.7%. Once the metabolic requirements for gas exchange were reduced during recovery from exercise, both hunting and domestic dogs increased respiratory evaporation [24]. In another study in a hot environment, heat loss from the respiratory tract was greatly increased, while conductance of heat through the tissue decreased, so that panting became increasingly important for maintaining a thermal balance [2]. This important route for heat loss probably made a significant contribution to the drop in body surface temperature after exercise in the current study.

Another explanation for the significant drop in body surface temperature at 120 AE could be an elevated basal body surface temperature before exercise (BE). Beagle dogs, as a representative of a small breed, have been perceived to be more excitable, anxious and fearful of a defined task than larger breeds [25]. Excitement contributes to an increase in muscle activity and associated alterations in blood flow [26]. Studies based on a group of baboons indicated that excitement increased the heart rate from 75 ± 4 to 208 ± 14 beats/min and mean arterial pressure from 92 ± 3 to 144 ± 9 mmHg (*p* < 0.01) [27]. In the literature, it is well documented that any type of positive stress caused by excitement induces an increase in core body temperature, leading to an elevated surface temperature in dogs [13,19]. A study on the emotional state of Beagles concluded that the dogs were excited not only by the expectation of a reward, but also by the realization that they themselves can control access to the reward [26]. Further studies are required in order to better understand how thermoregulatory processes are affected by behavior in Beagle dogs.

The surface temperatures obtained at the proximal limbs (upper forearm, shoulder, and thigh) were the most influenced by exercise on the treadmill. At rest, the upper hindlimb has been reported as one of the warmest parts of the body at rest in canines [16]. The fact that the UF, SH, and TH had the highest temperature during exercise could be associated with a higher concentration of blood flow and thermogenesis in the working muscles responsible for locomotion. Treadmill exercise especially affects activity of the hindlimb muscles, increasing blood flow [28]. Gluteal and hamstring muscles are the key muscle groups contributing to locomotion, thanks to their involvement in flexion of the hindlimb joints [29]. The forelimb muscles only exhibited increased activity in dogs trotting on a treadmill during the supporting and swing phases [30]. In the study presented by Rizzo et al. [6], Jack Russell Terrier/Miniature Pinscher mixed-breed dogs subjected to exercise on a treadmill had the highest body surface temperature at the internal thigh owing to a thinner hair coat or lack of coat in that region.

The coldest body regions in our study were located at the neck, croup, and back. It has been speculated that the neck region is cooler than other regions such as the ribs and inner thigh because the muscles are less active in that region [6]. Subcutaneous fat present in the back and croup area could also have an insulating effect that reduces heat loss through the skin. According to Ishioka et al. [31], the highest distribution of abdominal fat is in the area of vertebrae L3–5. Local differences in the length of coat could also be associated with variability in body surface temperature. Gunaratnam and Wilkinson [32] found that the area of the flanks had the most rapid growth rate of hair.

The major limitation of our study was associated with the low number of examined dogs. There were also large differences between the Beagle dogs in parameters including age, which is likely to have had an influence on thermal physiology and fitness. Our results cannot be extended to dogs of different breeds, or under different conditions. Moreover, as the order of the activity on the treadmill (walk, trot, and canter) was not randomized, we were not able to deduce the direct effect of the previous gait on temperature evolution. Randomization of the order of each gait would have allowed to study this effect.

## 5. Conclusions

Our study confirms that the body surface temperature of Beagle dogs is influenced by high-speed physical exercise on a treadmill owing to muscle activity and changes in blood flow. The proximal forelimb and hindlimb were the most influenced by exercise. The surface temperature at all ROIs does not remain significantly elevated after the cessation of exercise. IRT is a viable, non-invasive imaging modality that provides temperature data from specific body regions in canines during exercise. More research is now required to explore body surface temperature changes of different pure breeds in response to high-speed treadmill exercise.

## Figures and Tables

**Table 1:** Mean temperatures (±standard deviation) in seven regions of interest (ROIs): neck (NC), 5 min after treadmill exercises (5 AE), 15 min after treadmill exercise (15 AE), 30 min after treadmill exercise 30 AE, 45 min after treadmill exercise (45 AE), and 120 min after exercise (120 AE), as well as comparative results (ANOVA).

Time Points	Regions of Interest (ROIs)							*p*-Value
	NC	SH	UF	BC	CH	CR	TH	
BE	30.4 ± 1.2	31.0 ± 1.2	31.2 ± 1.2	30.1 ± 1.0	30.7 ± 1.0	29.7 ± 1.2	31.1 ± 1.1	0.057
AW	30.7 ± 0.8	31.3 ± 0.9	31.5 ± 1.0	30.2 ± 0.7	31.0 ± 0.9	29.9 ± 0.9	31.4 ± 1.0	0.001
AT	31.3 ± 0.7	31.5 ± 0.9	31.8 ± 0.9	30.6 ± 0.8	31.3 ± 0.8	30.1 ± 1.0	31.7 ± 1.0	0.001
AC	31.2 ± 1.3	32.0 ± 1.2	32.2 ± 1.2	30.9 ± 0.8	31.7 ± 1.0	30.6 ± 0.9	32.0 ± 1.1	0.013
JAE	31.3 ± 1.1	31.9 ± 1.0	32.2 ± 1.1	31.0 ± 1.1	31.7 ± 1.1	30.5 ± 1.0	32.1 ± 1.1	0.013
5 AE	30.9 ± 0.7	31.4 ± 0.8	31.7 ± 0.9	30.7 ± 0.8	31.2 ± 1.0	30.4 ± 0.8	31.7 ± 1.0	0.010
15 AE	30.9 ± 0.6	31.4 ± 0.7	31.8 ± 0.8	30.6 ± 0.8	31.3 ± 0.9	30.4 ± 0.9	31.7 ± 0.9	0.002
30 AE	30.7 ± 0.9	31.3 ± 0.8	31.7 ± 0.8	30.6 ± 0.7	31.3 ± 0.8	30.3 ± 0.9	31.6 ± 0.8	0.004
45 AE	30.7 ± 0.7	31.3 ± 0.5	31.6 ± 0.5	30.6 ± 0.5	31.2 ± 0.6	30.4 ± 0.7	31.5 ± 0.6	<0.001
120 AE	29.4 ± 0.7	30.1 ± 0.7	30.5 ± 0.7	29.2 ± 0.7	29.9 ± 0.8	28.7 ± 0.9	30.1 ± 0.6	<0.001
*p*-value	0.001	0.004	0.012	0.001	0.004	0.002	0.003	

**Table 2:** The result of multiple comparisons (*p*-value) between the temperatures (mean of all ROI) in subsequent stages of the study; significant differences were marked at the level of *p* < 0.05.

**ROI**	BE 30.6 °C (1.2 °C)	AW 30.9 °C (1.0 °C)	AT 31.2 °C (1.0 °C)	AC 31.5 °C (1.2 °C)	JAE 31.5 °C (1.2 °C)	5 AE 31.1 °C (1.0 °C)	15 AE 31.2 °C (0.9 °C)	30 AE 31.1 °C (0.9 °C)	45 AE 31.1 °C (0.7 °C)	120 AE 29.7 °C (0.9 °C)
BE		0.924	0.039	<0.001	<0.001	0.079	0.069	0.259	0.244	<0.001
AW			0.716	0.013	0.007	0.840	0.828	0.984	0.981	<0.001
AT				0.765	0.673	1.000	1.000	0.999	1.000	<0.001
AC					1.000	0.623	0.640	0.280	0.296	<0.001
JAE						0.522	0.539	0.208	0.221	<0.001
5 AE							1.000	1.000	1.000	<0.001
15 AE								1.000	1.000	<0.001
30 AE									1.000	<0.001
45 AE										<0.001
120 AE										

## Data Availability

The data presented in this study are available on request from the corresponding author. The data are not publicly available for privacy reasons.
